# Isolation and Identification of Type F Bovine Enterovirus from Clinical Cattle with Diarrhoea

**DOI:** 10.3390/v13112217

**Published:** 2021-11-04

**Authors:** Chengyuan Ji, Yao Zhang, Ruini Sun, Jiale Ma, Zihao Pan, Huochun Yao

**Affiliations:** 1 College of Veterinary Medicine, Nanjing Agricultural University, Nanjing 210095, China; 2019207042@njau.edu.cn (C.J.); zhangyao1387013@163.com (Y.Z.); sunyhchn@163.com (R.S.); jialema@njau.edu.cn (J.M.); panzihao@njau.edu.cn (Z.P.); 2 MOE Joint International Research Laboratory of Animal Health and Food Safety, Nanjing 210095, China

**Keywords:** bovine enterovirus, isolation, identification, genetic analysis, phylogenetic analysis

## Abstract

Recently, bovine enterovirus (BEV) has caused several respiratory and gastrointestinal diseases outbreaks in cattle. Monitoring the epidemiological and pathogenic characteristics of this virus is crucial to controlling its spread. We isolated a BEV strain with typical cytopathic effects from the faeces of cows with significant diarrhoeal symptoms in China and observed the viral particles within 20–30 nm through transmission electron microscopy. Then, we designated this strain as HB19-1 in this study. The multistep growth curves showed that the virus propagated well in the MDBK cells. Molecular genetic analysis of VP1 indicated that HB19-1 belonged to the BEV-F1 group. Although the challenged ICR mice did not exhibit typical disease symptoms in animal infection assay, we observed significant pathological damage in the lungs, intestines, and muscle tissues. In summary, we isolated a BEV strain HB19-1 causing severe diarrhoea in cattle and proposed reinforcing the epidemiological surveillance of this virus.

## 1. Introduction

The enterovirus genus within the picornaviridae includes nine enteroviruses (A–J) and three rhinoviruses (A–C) [1]. These viruses share similar characteristics and are the primary pathogens associated with respiratory and gastrointestinal diseases amongst humans and animals. Bovine enterovirus (BEV), similar to other species within the genus, are small and non-enveloped viruses with icosahedral and positive-stranded RNA genomes [2,3]. From the latest classification of enteroviruses, BEV is classified as EV-E and EV-F. EV-E has four subtypes (E1–E4), while EV-F has six (F1–F6). The overall length of the viral genome is around 7.5 kb. BEV encodes a single open reading frame (ORF) to produce a significant polyprotein precursor, undergoing a series of cleavage steps to produce four structural proteins (VP1, VP2, VP3, and VP4) and seven non-structural proteins (2A, 2B, 2C, 3A, 3B, 3C, and 3D) [4,5,6].

Most enteroviruses are well known for using the ‘canyon’ in the capsid proteins as binding sites for the cell surface receptors. VP1, VP2, and VP3 are the outer capsid proteins in enterovirus. Previous studies have suggested that VP1 may determine the ability of the virus to bind cells in enteroviruses and that several amino acid alterations in VP1 may yield changes in cytophilic and pathogenic properties [7,8,9]. Similar to other enteroviruses, the outer structural proteins of BEV form a ‘canyon’; thus, although the cellular receptor for BEV has not been identified, it is likely that VP1-based structural proteins likewise determine the virus phenotype [10].

The clinical signs of enterovirus infection amongst cattle vary from respiratory to intestinal diseases, reproductive diseases, and infertility [11,12,13,14]. The pathogenicity and virulence of BEV remain largely unknown. Previous studies have suggested that it is only of limited pathogenicity, as no clinically significant signs of BEV infection have been successfully replicated in calves [15]. However, as BEV isolates are increasingly identified from infected cattle, BEV pathogenicity and virulence have gained renewed attention, and their relationship with the disease has been intensively explored.

In this study, we report the isolation and characterisation of an EV-F1 strain of BEV from a herd of cattle in Hebei Province that had severe symptoms of diarrhoea. We used the ICR mouse infection model to perform an animal regression experiment and assess pathogenicity.

## 2. Materials and Methods

### 2.1. Cell Culture

Madin–Darby bovine kidney cells (MDBK; ATCC CCL-22) and baby hamster kidney cells (BHK-21; ATCC CCL10) were maintained in Dulbecco’s modified Eagle’s medium (DMEM) supplemented with 10% foetal bovine serum (FBS; Gibco) at 37 °C in a 5% CO_2_-enriched atmosphere.

### 2.2. Virus Isolation

In October 2019, a diarrhoea outbreak was reported in cattle on a dairy farm in Hebei, China. Some of these cattle had a slight fever, loss of appetite, and mild respiratory symptoms. In total, 18 faecal samples were subsequently collected for detection. These samples were diluted at 1:10 in a 10 mM phosphate-buffered saline (PBS) (pH 7.4) and clarified by low-speed centrifugation (3000× *g* for 10 min). Then, the supernatants were filtered through 0.22 μm pore membrane filters (Merck Millipore Ltd., Burlington, MA, USA). Virus isolation was performed on these specimens using MDBK cells. The monolayers of MDBK cells prepared in advance were washed with PBS; afterward, the filtered supernatant was inoculated onto the cells. The inoculum was discarded after 2 h of incubation with MDBK cells. The cells were washed with PBS before adding DMEM supplemented with 2% foetal bovine serum.

### 2.3. Library Construction and TruSeq Illumina Sequencing

The total RNAs from the faecal samples were extracted using a QIAamp Viral RNA Mini Kit (QIAGEN, Hilden, Germany). Viral RNA was extracted from infected-MDBK cells and was subjected to next-generation sequencing on an Illumina MiSeq platform (Shanghai Biozeron Technology Co., Ltd., Shanghai, China). The cDNA level was determined by a Qubit Fluorometer (Life Technologies, Seoul, Korea). Based on the manufacturer’s instructions (TruSeq RNA sample preparation kit), 230 ng cDNA samples were used to construct a library. Briefly, the nucleic acids were ultrasonicated to generate fragments of less than 500 bp. The DNA fragments were end-repaired using a T4 polynucleotide kinase. Then, the adaptors were ligated and loaded on a HiSeq 2000 (Illumina) for sequencing. The optimised sequences were spliced with multiple Kmer parameters using the ABySS v2.0.2 splicing software (http://www.bcgsc.ca/platform/bioinfo/software/abyss, accessed on 1 January 2020) to obtain the optimal assembly results. GapCloser v1.12 (https://sourceforge.net/projects/soapdenovo2/files/GapCloser/, accessed on 1 January 2020) software performed local inner hole filling and base correction on the assembly results.

When the samples were sequenced using the Illumina Hiseq sequencing platform, a specific percentage of raw data was of low quality, and the adapter sequences were removed from the raw sequencing reads to enable more accurate and reliable results of subsequent analysis. Bases containing non-AGCT at the 5′ end of the reads were removed, and the ends of the reads with a low sequencing quality (less than Q20) were trimmed before cutting. Reads containing up to 10% N were removed; small fragments of lengths less than 50 bp were discarded after the adapter, and quality trimming were removed.

### 2.4. Preparation of Anti-BEV Antibody and Indirect Immunofluorescence Assay (IFA)

Antiserum produced by mice was acquired as approved by the Institutional Animal Care and Ethics Committee of Nanjing Agricultural University. To generate the VP2 antibody against BEV, the VP2 coding region was amplified by RT-PCR using a forward primer (5′-GCTGATATCGGATCCGAATTCAGCCCTTCTGCTGAAGCCTG-3′) and a reverse primer (5′-GTGGTGGTGGTGGTGCTCGAGCTGCGCTACCGCTCTCCTC-3′). The VP2 PCR produced was double digested with EcoR I and Xho I and then ligated into the same digested pET-32a (+) expression vector (Novagen), resulting in plasmid pET32a-VP2 and BL21 (DE3). Escherichia coli cells were transformed with pET32a-VP2 and induced by 0.5 mM IPTG for 5 h. A HIS binding kit (Novagen) purified the recombinant VP2 protein. Finally, BABL/c mice were injected with the purified recombinant VP2 protein to generate a polyclonal antibody against the BEV VP2 protein (anti-BEV-VP2 PcAb).

IFAs tested the expression of viral proteins in BEV-infected MDBK cells, which were inoculated with HB19-1 at a multiplicity of infection (MOI) of 0.05. Post-incubation, the cells were fixed with 4% paraformaldehyde, incubated with anti-BEV-VP2 PcAb (diluted 1:100) for 1 h at 37 °C, and then incubated with a FITC-conjugated secondary antibody for 1 h at 37 °C. Cellular nuclei were stained with 10 μg/mL DAPI (Roche) for 5 min, and the samples were examined by fluorescence microscopy (Zeiss, Oberkochen, Germany).

### 2.5. Electron Microscopy Observation and Plaque Assay

After freezing and thawing the virus cultures thrice, the supernatant was removed by centrifugation at 8000 rpm for 30 min at 4 °C; after centrifugation at 25,000 rpm for 3 h, the supernatant was discarded. Finally, the precipitate was resuspended in 100 uL PBS, and the sample was incubated with 2% phosphotungstic acid. The viruses were observed using electron microscopy (HITACHI HT7700).

The MDBK cells grown in six-well plates were used to perform the viral plaque assays. The viral samples were serially 10-fold diluted in DMEM. Each virus dilution was inoculated onto a monolayer of MDBK cells with a dose of 500 µL. Following incubation for 1 h, the cells were overlaid with a mixture of DMEM containing 1% low-melting agarose (Cambrex, Rockland, ME, USA) and 2% FBS and incubated for 3 days at 37 °C, 5% CO_2_. The medium was then carefully removed from the plates, and the cells were stained with 3–4 mL of staining solution, which comprised 0.5% crystal violet and 25% formaldehyde solution, for 15 min. Remarkably, visible plaques were observed.

### 2.6. TCID50 Titration of Virus Isolates and Growth Curve

Titration of TCID50 for HB19-1 isolates was performed using 96-well plates. Briefly, the viruses were diluted at 10× serial dilutions and used to infect the wells for each dilution. Then, 48 h post-inoculation, the cytopathic effects on the MDBK cells were observed and counted, and TCID50 was calculated following a standard procedure. The virus titres (TCID50) for each time point were assessed and calculated based on the Reed–Muench method [16] to describe the viral growth kinetics. The virus isolates (P5) were briefly inoculated into the MDBK cells growing in the six-well plates at an MOI of 0.05. After incubation at 37 °C for 1 h, the cells were washed twice with PBS. Two hundred microliters of MDBK cell supernatants were harvested at 12, 24, 36, 48, and 72 hpi and stored at −70 °C. After measuring the mean titres of three independent measurements at each time and point, the growth curves were determined.

### 2.7. Sequence Alignments and Phylogenetic Analysis

Differences in the sequence of this isolate and all other BEV strains available from GenBank were analysed using the MegAlign program with the DNAstar 7.0 software. The information regarding the reference BEV strains was downloaded from GenBank. Phylogenetic analyses were performed based on the complete genome and VP1 gene. Amino acid sequences were deduced from nucleotide sequences using the EditSeq module of the DNAstar software package. Multiple sequence alignments and phylogenetic trees were constructed through the neighbour-joining method using Molecular Evolutionary Genetics Analysis (MEGA, Auckland, New Zealand, version 7.0) software, and a bootstrap resampling analysis of 1000 replicates was performed.

### 2.8. Animal Experiments

Ethics approval of animal experiments was as described for the antiserum produced in mice. Pregnant ICR mice were obtained at Yangzhou University. The mice were maintained in the Laboratory Animal Facility of Nanjing Agriculture University. The animals had free access to food and water and were kept in a temperature-controlled room (24 ± 0.5 °C). Twenty 3-day-old pups were divided into two groups, with each cage litter containing a dam and ten pups. In experimental groups, 3-day-old ICR mice were administered an intraperitoneal injection of 50 uL viruses at 10^7^ TCID50. The negative control group was injected with DMEM. Clinical symptoms were monitored daily.

### 2.9. The Replication Kinetics of Virus in ICR Mice

The mice were dissected, and their heart, liver, spleen, lungs, kidneys, brain, and hind limb muscles were collected 3-, 7-, and 14 days post-infection to monitor the changes in HB19-1 viral load in vivo. We designed the probes in the BEV 5′ UTR region to establish a real-time quantitative polymerase chain reaction method. BEV was detected by Taq-Man qPCR (F, 5′-CGTGAATGCTGCTAATCC-3′; Probe, 5′-TGCGCACAAWCCAGTGTTGCTRCGT-3′; R, 5′-RACGGYGTACCGAAAGTAG-3′). The total RNAs from the tissues were isolated using the Total RNA Kit II (QIAGEN, Hilden Germany). All operations followed the animal tissue protocol in the instructions. The total RNAs extracted from 20 mg of tissues were reverse-transcribed into cDNA using the HiScript^®^ II 1st Strand cDNA Synthesis Kit (Vazyme, Nanjing, China), quantified by Taq-Man qPCR.

### 2.10. Histopathology of HB19-1-Infected ICR Mice

The heart, liver, spleen, lung, kidney, brain, intestine, and muscle tissues of ICR mice after 3 days infection were collected and placed in a 4% (*v*/*v*) paraformaldehyde fixative solution for histopathological analysis. The paraffin blocks embedded with fixed tissues or organs were serially cut into 5 mm sections and mounted on glass slides. The pathological sections stained with haematoxylin-eosin (HE) were then performed in microscopic examination to evaluate the tissues’ pathological damage.

### 2.11. Retrospective Research

To investigate the prevalence of BEV on the cattle farm, 78 cattle droppings were obtained again. Based on the Taq-Man real-time qPCR method, we tested these 78 cattle faeces for BEV. For the serological epidemiological survey, 50 bovine serum samples were obtained. The BEV-specific antibody titres were determined by IFA as described previously. Briefly, MDBK cells were inoculated with BEV at an MOI of 1 for 12 h. IFA was performed using the 50 serum samples with 4-fold serial dilutions. All samples were assessed in triplicate.

### 2.12. Statistical Analysis

Summary statistics were calculated to assess the overall data quality. All data were processed using the GraphPad Prism software (version 8.0). In vitro experiments were performed independently at least thrice, while the *t*-test evaluated the statistical significance of the virus titre. Statistical significance was assessed at *p* values of less than 0.05, 0.01, or 0.001.

## 3. Results

### 3.1. Isolation and Identification of the BEV Strain

**Figure 1:**
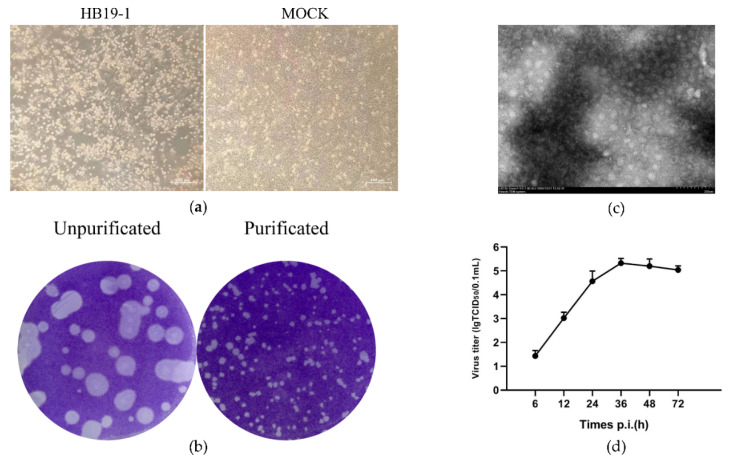
Isolation and identification of BEV HB19-1. CPE, plaque purification, electron microscopy, and growth curve of BEV HB19-1 in MDBK cells: (**a**) CPE caused by BEV HB19-1 in MDBK cells was characterised at 24 hpi; (**b**) plaques of impurified and purified BEV HB19-1 strain; (**c**) electron microscopy of purified BEV HB19-1 virions; (**d**) growth curve of BEV HB19-1 in MDBK cells.

Bovine diarrhoeic faecal specimens were examined by RT-PCR using the specific primers to detect the significant pathogens, which depicted negative results for BVDV, BRV, BCoV, and BHV. However, the MDBK cells inoculated with the above specimens indicated a typical cytopathic effect as early as 12 h. Initially, the cells began to stack and round, and 24–36 h after inoculation, most of the infected cells were detached off the culture dish. We subsequently performed at least five generations of cultures containing the inoculum. We observed similar cytopathic effects in each passaging, signifying that the infection of the potential pathogens caused the CPE in the inoculum (Figure 1a). The whole genome of the inoculum was sequenced, and several fragments showed high-sequence identity with BEV, and no other viral matches were detected. Subsequently, the whole genome of a BEV strain was assembled and analysed (GenBank accession no. MW468092), revealing that the pathogen was a BEV isolate and thus designated as HB19-1. The plaques generated by BEV HB19-1 in MDBK cells were regularly shaped with distinct edges. Further assays indicated that the plaques were approximately 1–2 mm in diameter after three purifications (Figure 1b).

### 3.2. Plaque Purification and Growth Curve

To identify the potential virus, the cell supernatant was processed for electron microscopic observation. Virus particles with 20–30 nm diameter were observed in the infected cells (Figure 1c). The multistep growth curves of the HB19-1 strain were further analysed using MDBK cells. As illustrated in Figure 1d, the viral load exhibited a gradual increase from 6 hpi and reached a peak titre of ∼10^6^ TCID50 at 36 hpi. The titres then gradually decreased, reaching a titre of ∼105 TCID50 at 72 hpi. These results revealed that a BEV strain was successfully isolated using the MDBK cell.

### 3.3. Whole-Genome and Phylogenetic Analyses

The length of the complete genome of BEV HB19-1 was 7357 nt, including an 807 nt 5′ UTR sequence, a 6504 nt polyprotein gene, and a 46 nt 3′ UTR sequence. The ORF of HB19-1 was located between nucleotides 808 and 7312 and encoded a potential polyprotein of 2167 amino acid residues with a calculated molecular mass of approximately 241.7 kDa. Sequence alignments of the deduced amino acids of the polyprotein were compared with the representative reference BEV strains to determine the genetic relationships and attributes of BEV HB19-1. The genomic data of BEV strains in the GenBank database were downloaded for comparative genomic and phylogenetic analysis. The results indicated that the BEV HB19-1 shared 66.9–79.4% of sequence identity with the other strains in the whole genome level, sharing the highest identity (79.4%) with the IL-alpaca strain. Furthermore, the comparison of the amino acid sequence for the encoded proteins was performed. The HB19-1 strain determined the sequence identities in the ranges of 65.6–91.0% and 77.8–96.0% in the structural and non-structural polyproteins, respectively (Table 1).

**Figure 2:**
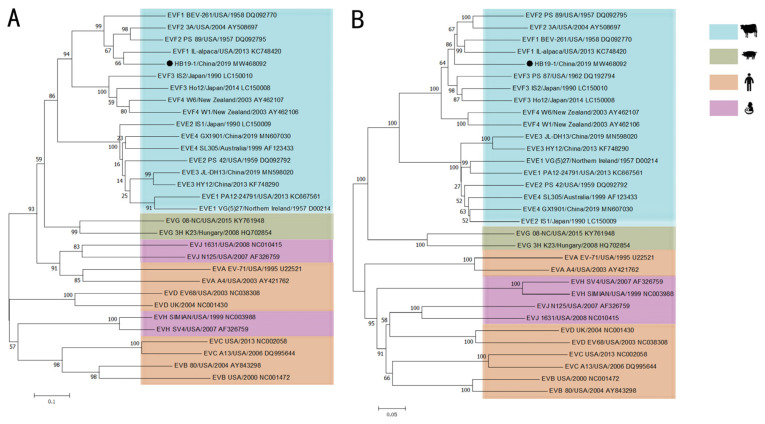
Phylogenetic analyses of the BEV HB19-1 strain. Phylogenetic analyses were based on the amino acid sequences. Phylogenetic trees were constructed using the neighbour-joining method with 1000 bootstrap replicates in the MEGA 7.0 software. The scale bars indicate the amino acid or nucleotide substitutions at each site: (**A**) VP1 of the BEV HB19-1 strain; (**B**) the polyprotein of the BEV HB19-1 strain.

EV classification was solely based on the sequence identities of the VP1 gene, and the phylogenetic tree based on the VP1 gene amino acid sequences signified that the HB19-1 strain was clustered into the EV-F1 subtype, most closely associated with the 1L-alpaca/USA/2013 strain. Additionally, both phylogenetic trees of VP1 and polyprotein showed that the BEV strains had a closer genetic relationship with the porcine enterovirus (PEV) strains (Figure 2).

### 3.4. Replication Kinetics and Histopathology of HB19-1-Infected ICR Mice

**Figure 3:**
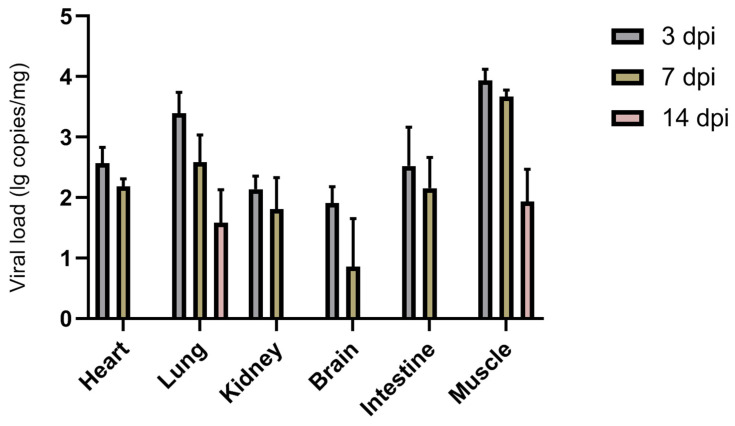
Replication kinetics of HB19-1-infected mice. Detection and quantification of the BEV HB19-1 strain in ICR mice. Heart, liver, spleen, lungs, kidneys, brain, intestines, and muscle tissues of the BEV HB19-1-infected mice were obtained in triplicate. The total cellular RNA was extracted from the tissues and then analysed by RT-qPCR. The data are expressed as mean ± SD of three samples. Error bars represent the SD from triplicate within an experiment.

BEV HB19-1 was inoculated into healthy 3-day-old neonatal ICR mice to determine the pathogenicity of the isolated HB19-1 strain. No significant clinical signs were observed in either the inoculation group or the control group. To explore further the viral antigen distribution within infected mice, diverse tissue samples were obtained for viral RNA detection (Figure 3). Viral RNA was detectable in the heart, lungs, kidneys, brain, intestines, and muscle tissues but not in the liver and spleen. Viral load was highest at 3 dpi and gradually decreased with increasing time. Notably, HB19-1 replicated best in lungs and muscle tissues and did not exhibit high viral loads in the intestines. These results suggested that HB19-1 could replicate relatively well in ICR mice.

**Figure 4:**
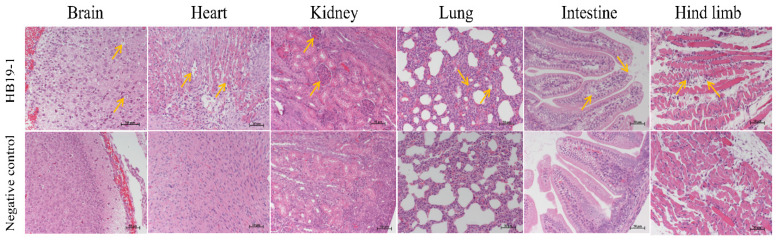
Histopathology analysis of HB19-1-infected ICR mice after 3 days infection. Heart, liver, spleen, lungs, kidneys, brain, intestines, and muscle tissues of the BEV HB19-1-infected mice were obtained. At the indicated time points post-infection, the paraffin sections of tissues were stained with haematoxylin–eosin (HE) and observed under a light microscope. All observations were performed at 200× magnification. Black arrows indicate the typical lesion.

HE-stained pathological sections were observed under light microscopy to assess the extent of histopathological damage. In agreement with the qRT-PCR results, ICR mice’s heart, lungs, kidneys, brain, intestines, and muscle tissues showed significant lesions after BEV HB19-1 infection (Figure 4). Sections of corresponding tissues in the negative control group were typical. Accordingly, the heart sections of vacuolar degeneration were present in the heart. In addition, alveolar wall interstitial thickening and scattering of red blood cells in the alveolar space were observed.

Furthermore, mild glomerular enlargement and congestion were observed in the kidneys. The pathological damage in the brain was cell degeneration and vacuolation. Moreover, the vacuolar changes in epithelial cells and scattering of the intestinal villi were observed, indicating that the small intestinal mucosal structure was destroyed. Otherwise, inflammatory cell infiltration, muscle fibre disorder, or partial dissolution were observed in the muscle tissues.

**Figure 5:**
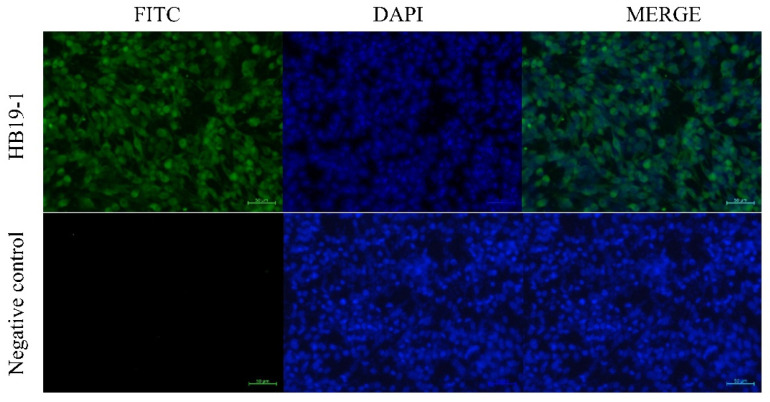
BHK-21 cells inoculated with tissues obtained from HB19-1-infected ICR mice showed many BEV-positive cells by IFA at 36 hpi.

Furthermore, the virus was reisolated from the BEV-infected tissues with evident CPE, and several BEV-positive cells were observed in IFA with the antiserum specific for BEV VP2 at 36 hpi (Figure 5).

### 3.5. Epidemiological Investigation of BEV Infection in the Farm

To assess the prevalence rate of BEV infection in the farm isolated the BEV strain HB19-1, 50 samples (approximately 5% of the farm’s cows), 17 with clinical diarrhoea and 33 asymptomatic, were collected and investigated by qRT-PCR. Of the 50 samples, 23 were positive for BEV (46.00%), with 16 of the diarrhoea samples (94.12%) and 7 of the asymptomatic samples (21.21%) positive. Meanwhile, for seroepidemiological investigation, 50 cows’ serum samples were collected and investigated by IFA. A high BEV-positive rate (84.00%, 42/50) was observed in collected sera. These results revealed that BEV infection was highly prevalent in cow herds and that the proportion of BEV infection in diarrhoea samples was higher than in asymptomatic samples.

## 4. Discussion

Recent BEV infection outbreaks have led to diarrhoea-like illness in cattle, suggesting that the virus may pose a threat to intestinal health in cattle. This study reports the isolation of a bovine enterovirus strain HB19-1 from a cattle farm in Hebei province. The HB19-1 isolate was from calves with severe diarrhoea. Based on clinical signs, BCV, BRV, or BVDV were allegedly involved in this case, but only BEV was isolated. The final pathogen was subsequently identified as a BEV by whole-genome sequencing. For accurate classification of the isolated virus, phylogenetic analysis was performed on three logical levels (family, genus, and species). The BEV strain HB19-1 (MW468092) was classified as Picornaviridae under the enterovirus genus and BEV-F1 species. Although some reports have overlooked BEV pathogenicity, increasing reports of BEV isolation from respiratory and diarrhoeal cattle suggest the relevance of BEV to these diseases. The detection and isolation of BEV HB19-1 in dairy cattle with severe diarrhoeal disease indicate that HB19-1 may play a vital role as a pathogen.

Although the best approach to determine microbial pathogenicity is to infect the animal species with isolated pathogens, this system is not suitable for studying those isolated from large animals, primarily due to the lack of animals with the required background and the related costs of the experiment. Animal model systems remain the most effective means of studying microbial pathogenicity and pathogenesis, and the mouse infection model has been extensively used in studying viral infections. For human enteroviruses, mice, hamsters, and various transgenic mice have been evaluated for pathogenic mechanisms and vaccine efficacy [17,18,19]. However, no animal model has been widely recognised and accepted for assessing BEV; hence, even in neonatal mice, we have not suitably reproduced the typical clinical signs of BEV.

Furthermore, enterovirus can multiply stably and efficiently in the host and remain in the environment for a long time after mass elimination from the host. A previous large-scale surveillance study suggests that enterovirus infection may be associated with diarrhoea in yaks in some areas. The prevalence of this virus was significantly higher in diarrhoeal samples than healthy yaks, suggesting a potential correlation with the clinical signs of diarrhoea [20]. The epidemiological investigation in this study indicated that the rate of BEV positivity in diarrhoeal samples was much higher than in asymptomatic samples, suggesting that BEV is most likely to cause bovine intestinal disease and therefore cannot be ignored in controlling bovine diarrhoea. Furthermore, as no other diarrhoea-like viruses were detected in these faeces, it is unclear whether mixed or secondary infection exists with other bovine pathogens [21,22,23]. Notably, BEV may have played a role in transmitting to other animals, such as yak, sheep, goats, and horses, indicating that this virus has the potential to spread nationally and expand its host range [20,24,25,26,27]. Unfortunately, there is a lack of epidemiological investigation of BEV in China. In response, we recommend implementing continuous surveillance of BEV infection in animals to control the circulation of this potentially dangerous virus.

## 5. Conclusions

This study determined the pathogenic characteristics of EV-F strain HB19-1 using various assays, including CPE, electron microscopy, replication kinetics, and animal experiments. The whole-genome analysis and phylogenetic trees indicated that strain HB19-1 was classified into EV-F1 genotype. Thus, this study provides a reference for the biological characterisation, evolution, and pathogenicity of EV-F.

## Figures and Tables

**Table 1:** Nucleotide and amino acid sequences and identity analysis of HB19-1 and the other BEV strains.

		HB19-1 Identity (%)					
Strains	GenBank Accession No.	Complete Genome	Structural Protein		Non-Structural Protein		Sub-Genotype
		nt	nt	aa	nt	aa	
VG(5)27	D00214	67.2	61.4	66.5	68.4	77.8	EV-E1
PA12-24791	KC667561	67.4	62.0	66.4	68.7	78.0	EV-E1
IS1	LC150009	67.9	63.0	65.9	69.2	78.4	EV-E2
PS42	DQ092792	67.8	62.6	66.6	69.5	78.7	EV-E2
JL-DH13	MN598020	66.9	62.6	66.0	68.1	77.8	EV-E3
HY12	KF748290	67.2	62.3	65.9	69.0	78.2	EV-E3
SL305	AF123433	67.3	62.0	65.6	69.2	78.2	EV-E4
GX1901	MN607030	67.9	63.0	66.1	69.6	78.2	EV-E4
BEV-261	DQ092770	78.9	75.3	90.6	80.4	94.5	EV-F1
IL-alpaca	KC748420	79.4	75.5	91.0	80.8	95.5	EV-F1
3A	AY508697	78.3	73.1	86.9	79.9	93.9	EV-F2
PS89	DQ092795	77.8	73.1	86.8	79.9	94.0	EV-F2
Ho12	LC150008	78.3	70.7	79.2	81.7	95.7	EV-F3
IS2	LC150010	77.4	69.0	79.8	81.2	96.0	EV-F3
PS87	DQ092794	77.8	70.0	79.4	81.4	95.6	EV-F3
W1	AY462106	75.2	69.9	79.6	77.1	90.7	EV-F4
W6	AY462107	75.5	69.9	77.6	78.1	91.9	EV-F4

## Data Availability

Data are contained within the article.

## References

[1] Lefkowitz E.J., Dempsey D.M., Hendrickson R., Orton R.J., Siddell S.G., Smith D.B. (2018). Virus taxonomy: The database of the International Committee on Taxonomy of Viruses (ICTV). Nucleic Acids Res..

[2] Zell R., Krumbholz A., Dauber M., Hoey E., Wutzler P. (2006). Molecular-based reclassification of the bovine enteroviruses. J. Gen. Virol..

[3] Hyypi T., Hovi T., Knowles N.J., Stanway G. (1997). Classification of enteroviruses based on molecular and biological properties. J. Gen. Virol..

[4] Ng Q., He F., Kwang J. (2015). Recent Progress towards Novel EV71 Anti-Therapeutics and Vaccines. Viruses.

[5] Zhou F., Kong F., Wang B., McPHIE K., Gilbert G.L., Dwyer D.E. (2011). Molecular characterization of enterovirus 71 and coxsackievirus A16 using the 5′ untranslated region and VP1 region. J. Med. Microbiol..

[6] Earle J.A., Skuce R.A., Fleming C.S., Hoey E.M., Martin S.J. (1988). The complete nucleotide sequence of a bovine entero-virus. J. Gen. Virol..

[7] Kataoka C., Suzuki T., Kotani O., Iwata-Yoshikawa N., Nagata N., Ami Y., Wakita T., Nishimura Y., Shimizu H. (2015). The Role of VP1 Amino Acid Residue 145 of Enterovirus 71 in Viral Fitness and Pathogenesis in a Cynomolgus Monkey Model. PLOS Pathog..

[8] Caine E.A., Moncla L.H., Ronderos M.D., Friedrich T.C., Osorio J.E. (2016). A Single Mutation in the VP1 of Enterovirus 71 Is Responsible for Increased Virulence and Neurotropism in Adult Interferon-Deficient Mice. J. Virol..

[9] Lin J.-Y., Shih S.-R. (2014). Cell and tissue tropism of enterovirus 71 and other enteroviruses infections. J. Biomed. Sci..

[10] Smyth M., Symonds A., Brazinova S., Martin J. (2002). Bovine enterovirus as an oncolytic virus: Foetal calf serum facilitates its infection of human cells. Int. J. Mol. Med..

[11] Zhu L., Xing Z., Gai X., Li S., San Z., Wang X. (2014). Identification of a Novel Enterovirus E Isolates HY12 from Cattle with Severe Respiratory and Enteric Diseases. PLoS ONE.

[12] Li Y., Chang J., Wang Q., Yu L. (2012). Isolation of two Chinese bovine enteroviruses and sequence analysis of their complete genomes. Arch. Virol..

[13] McCarthy F., Smith G., Mattick J. (1999). Molecular characterisation of Australian bovine enteroviruses. Vet. Microbiol..

[14] Shaukat S., Angez M., Alam M.M., Sharif S., Khurshid A., Malik F., Rana M.S., Mahmood T., Zaidi S.S.Z. (2012). Molecular Identification and Characterization of a New Type of Bovine Enterovirus. Appl. Environ. Microbiol..

[15] Blas-Machado U., Saliki J., Sanchez S., Brown C.C., Zhang J., Keys D., Woolums A., Harvey S.B. (2011). Pathogenesis of a Bovine Enterovirus-1 Isolate in Experimentally Infected Calves. Vet. Pathol..

[16] Stanic M. (1963). A simplification of the estimation of the 50 percent endpoints according to the Reed and Muench method. Pathol. Microbiol..

[17] Wu Y., Qu Z., Xiong R., Yang Y., Liu S., Nie J., Liang C., Huang W., Wang Y., Fan C. (2021). A practical method for evaluating the in vivo efficacy of EVA-71 vaccine using a hSCARB2 knock-in mouse model. Emerg. Microbes Infect..

[18] Dong Z., Liu Z.-W., Chen R., Wen X.-J., Ji J., Zheng X.-X., Zhao L., Wang Z.-Y., Wen H.-L. (2019). The untranslated regions of EV-A71 contribute to its pathogenicity and virulence. Virus Res..

[19] Yu P., Bao L., Xu L., Linlin B., Lv Q., Deng W., Xu Y., Qin C. (2017). Neurotropism In Vitro and Mouse Models of Severe and Mild Infection with Clinical Strains of Enterovirus 71. Viruses.

[20] He H., Tang C., Chen X., Yue H., Ren Y., Liu Y., Zhang B. (2016). Isolation and characterization of a new enterovirus F in yak feces in the Qinghai-Tibetan Plateau. Arch. Virol..

[21] Zhang M., Hill J.E., Alexander T.W., Huang Y. (2021). The nasal viromes of cattle on arrival at western Canadian feedlots and their relationship to development of bovine respiratory disease. Transbound. Emerg. Dis..

[22] Dunne H.W., Ajinkya S.M., Bubash G.R., Griel L.C. (1973). Parainfluenza-3 and bovine enteroviruses as possible important causative factors in bovine abortion. Am. J. Veter. Res..

[23] Mitra N., Cernicchiaro N., Torres S., Natalia C., Hause B.M. (2016). Metagenomic characterization of the virome associated with bovine respiratory disease in feedlot cattle identified novel viruses and suggests an etiologic role for influenza D virus. J. Gen. Virol..

[24] Zheng T. (2007). Characterisation of two enteroviruses isolated from Australian brushtail possums (Trichosurus vulpecula) in New Zealand. Arch. Virol..

[25] Jimenez-Clavero M.A., Escribano-Romero E., Mansilla C., Gomez N., Cordoba L., Roblas N., Ponz F., Ley V., Saiz J.C. (2005). Survey of bovine enterovirus in biological and environmental samples by a highly sensitive real-time reverse transcrip-tion-pcr. Appl. Environ. Microbiol..

[26] Hamblin C., Knowles N., Hedger R. (1985). Isolation and identification of bovid enteroviruses from free-living wild animals in Botswana. Veter. Rec..

[27] Boros A., Pankovics P., Knowles N.J., Reuter G. (2012). Natural interspecies recombinant bovine/porcine enterovirus in sheep. J. Gen. Virol..

